# Advances in modelling of biomimetic fluid flow at different scales

**DOI:** 10.1186/1556-276X-6-344

**Published:** 2011-04-15

**Authors:** Sujoy Kumar Saha, Gian Piero Celata

**Affiliations:** 1 Mechanical Engineering Department, Bengal Engineering and Science University, Shibpur, Howrah, West Bengal 711 103, India; 2 ENEA Casaccia Research Centre, Institute of Thermal Fluid Dynamics, Office Building F-20, Via Anguillarese 301, S. M. Galeria, Rome 00123, Italy

## Abstract

The biomimetic flow at different scales has been discussed at length. The need of looking into the biological surfaces and morphologies and both geometrical and physical similarities to imitate the technological products and processes has been emphasized. The complex fluid flow and heat transfer problems, the fluid-interface and the physics involved at multiscale and macro-, meso-, micro- and nano-scales have been discussed. The flow and heat transfer simulation is done by various CFD solvers including Navier-Stokes and energy equations, lattice Boltzmann method and molecular dynamics method. Combined continuum-molecular dynamics method is also reviewed.

## Introduction

**Figure 1:**
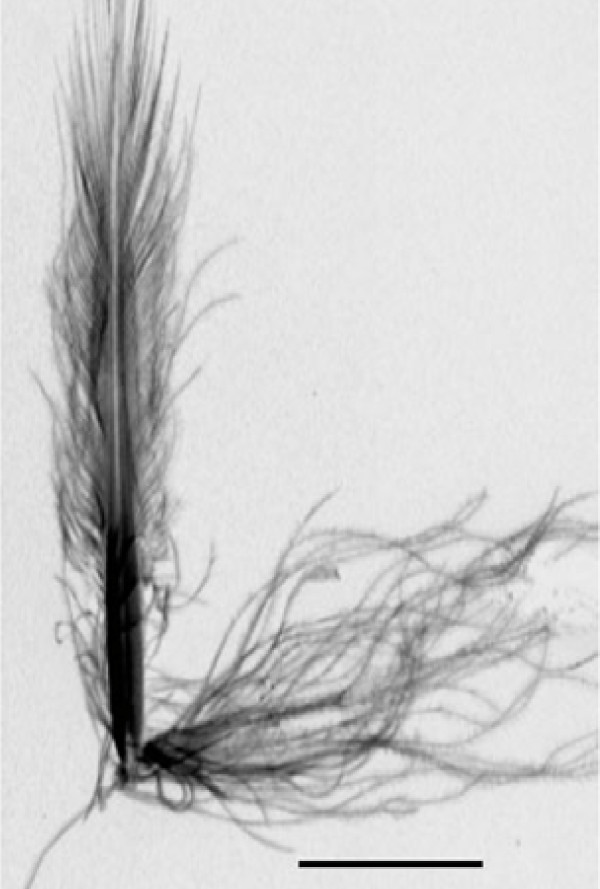
**Feather of a penguin to stay warm naturally in a cold climate**. (From [4]).

**Figure 2:**
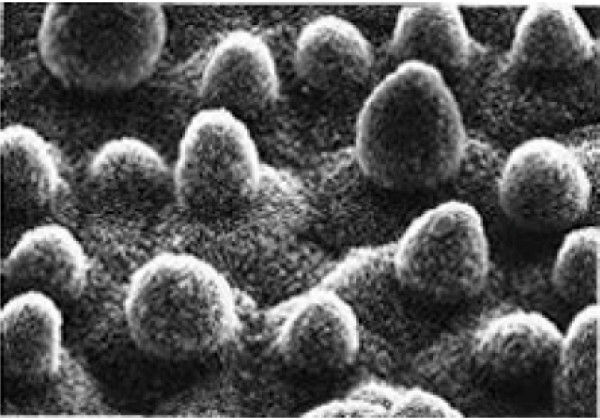
**The epidermal structure at the heart of the lotus effect**. (From [11]).

Human knowledge is getting enriched from the four billion years' worth of R & D in the natural world of plants and animals and other lower level living creatures and microorganisms, which have evolved through the ages to nicely adapt to the environment. Man has now drawn his attention to soil creatures like earthworms, dung beetle, sea animals like shark and plants and trees like lotus leaf and pastes like termites. In the nature, we see examples of effortless and efficient non-sticking movement in mud or moist soil, high-speed swimming aided by built-in drag-reduction mechanism, water repellant contaminant-free surface cleaning mechanism and natural ventilation and air conditioning, [1-8]. By nature, feather of the penguin shows staying warm naturally, Figure 1[4]. The leaf of the lotus is hydrophobic to the extent that water running across the surface of the leaf retains particles of dirt caused by a thick layer of wax on the surface and the sculpture of that surface, Figure 2[9-11]. This forces the droplets of water to remain more or less spherical when in contact with the leaf, and reduces the tendency of other contaminants to stick to the leaf. It has been proved that water repellency causes an almost complete surface purification (self-cleaning effect): contaminating particles are picked up by water droplets or they adhere to the surface of the droplets and are then removed with the droplets as they roll off the leaves. This characteristic has been utilized in exterior-quality paint, 'Lotusan', which makes surfaces self-cleaning. Hooks occur in nature as a vast array of designs and in a diversity of animals and plants. The commercial application of this technology of 'Nature' can be found in Velcro [5] having the cheapest and most reliable bur hook-substrate combination. There are now thousands of patents quoting Velcro. This is how the subject of biomimetics has developed. Biomimetics is the application and abstraction of biological methods, systems and good designs found in nature to the study and design of efficient and sustainable engineering systems and modern technology. The transfer of technology between lifeforms and manufactures is desirable because evolutionary pressure typically forces living organisms, including fauna and flora, to become highly optimized and efficient. Generally there are three areas in biology after which technological solutions can be modelled.

• Replicating natural manufacturing methods as in the production of chemical compounds by plants and animals.

• Mimicking mechanisms found in nature such as Velcro and Gecko tape.

• Imitating organizational principles from social behaviour of organisms like ants, bees and microorganisms.

Russia has developed a systematic means for integrating the natural knowledge into humankind's technology using 'Teoriya Resheniya Izobretatelskikh Zadatch (TRIZ)', i.e. the theory of inventive problem solving, which provides an objective framework based on functionality for accessing solutions from other technologies and sciences. TRIZ also prevents waste of time trying to find a solution where none exists. The four main tools of TRIZ are a knowledge database arranged by function, analysis of the technical barriers to progress (contradictions), the way technology develops (ideality) and the maximization of resource usage. The biology-based technology 'Biomimetics' suggests new approaches resulting in patents and some into production:

• Strain gauging based on receptors in insects [7],

• Deployable structures based on flowers and leaves [12],

• Tough ceramics based on mother-of-pearl [13],

• Drag reduction based on dermal riblets on shark skin [14],

• Tough composites based on fibre orientations in wood [15],

• Underwater glues based on mussel adhesive [16],

• Flight mechanisms based on insect flight [2],

• Extrusion technology based on the spinneret of the spider [3],

• Self-cleaning surfaces based on the surface of the lotus leaf [17].

The importance of Biomimetics will increase as the incidence of genetic manipulation increases and the genetic manufacturing is developed. In the result, the area between living and non-living materials, where biology interacts with engineering, e.g. bioengineering and biomechatronics, is benefited.

There are innumerable examples of interactions with the environment and balanced and efficient heat, mass, momentum and species transfer through the microstructures in the fluid flow in the manifested living world of plants, animals and other living creatures. Biomimetics involve mimicking these interactions across the functional surfaces with the surrounding environments in the technological design. The physical nature is numerically modelled and simulated using computational fluid dynamics (CFD).

Geometrical analogy as well as physical similarity is to be studied to design technological functional surfaces imitating microstructural and biological functional surface morphologies. CFD at micro- or meso-scales and other numerical methodologies are necessary for this [18-24].

The meso- and micro-scale methods are also being developed in parallel with the continuum theory-based conventional CFD techniques-using finite volume method (FVM) and finite element method (FEM). In the mesoscopic lattice Boltzmann method (LBM), fluid flow is simulated by tracking the development of distribution functions of assemblies of molecules. It is difficult to capture the interfacial dynamics, which is essential for multiphase flow, at the macroscopic level. LBM captures the interaction of fluid particles and is, therefore, helpful for multiphase flow with phase segregation and surface tension. Also, the LBM is computationally more efficient than molecular dynamics (MD) method since it does not track individual molecules; the solution algorithm is explicit, easy to implement and parallel computation can be done. Micro/nano-scale simulations in micro/nano-scale geometries and micro time scales are done in MD method and direct simulation of Monte Carlo (DSMD) method. Coupled macro-scale simulation is being done using high performance computer (HPC). This article makes a review of the advances in multiscale biomimetic fluid flow modelling and simulation of difficult physics problems with complex biological interfaces.

### Macroscopic biomimetic flow modelling

**Figure 3:**
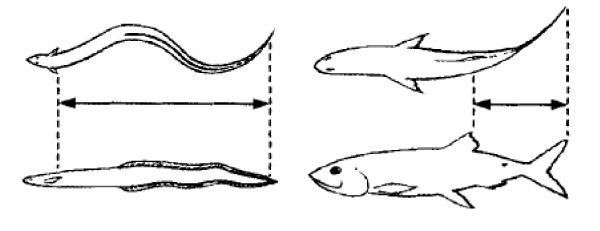
**The modes of swimming of fishes**. **(a) **The anguilliform motion of an eel. **(b) **The carangiform motion of a tuna. (From [25]).

The locomotion, power and manoeuvring of aquatic animals like swimming fish having superior and efficient utilization of propulsion through a rhythmic unsteady motion of the body and fin resulting in unsteady flow control has been engineered for the transportation in the underwater vehicles. The fish senses and manipulates large-scale vortices and repositions the vortices through tail motion. The timing of formation and shedding of vortices are important. CFD application by mimicking the swimming of fish and underwater dolphin kicking has been utilized to understand active drag and propulsive net thrust and this has resulted in better sailing performance, Olympic ski jumping, Formula 1 racing, Speedo's new Fastskin FSII swimsuit and an optimal kick profile in swim starts and turns. The undulatory propulsion in aquatic vertebrates is achieved by sending alternating waves down the body towards the tip of the tail and causing sinusoidal oscillation of the body, a jet in the wake and a forward thrust. Two modes of propulsive technique utilized by fish are anguilliform and carangiform, Figure 3[25]. The carangiform mode is also termed as 'lunate-tail swimming propulsion'.

The unsteady incompressible Navier-Stokes equations of turbulent flow are solved in the simulation by applying the Reynolds-averaged Navier-Stokes (RANS) equations with usual boundary conditions to obtain the fluctuating velocity fields. The equations in Cartesian tensor form are:(1)(2)(3)(4)(5)(6)(7)

where *x *and *u *are Cartesian coordinates and velocities, respectively, and *t *is time. Velocity u, density ρ, viscosity μ and other solution variables represent ensemble-averaged (or time-averaged) values. Reynolds stress,  is modelled and related to the mean velocity gradients by Boussinesq hypothesis. *k *is the turbulence kinetic energy, ε the kinetic energy dissipation rate and μ_t _the turbulent viscosity. *C *is constant, σ the Prandtl number. *G*_k _represents the generation of turbulence kinetic energy due to the mean velocity gradients. μ_t _is the turbulent viscosity.

The turbulent flow induced by the fish-tail oscillation is characterized by fluctuating velocity fields. The instantaneous governing equations are time averaged to reduce the computational time and complexity which is done in the form of turbulence models like the semi-empirical *k*-ε work-horse turbulence model for practical engineering flow calculations.

To calculate the flow field using the dynamic mesh, the integral form of the conservation equation for a general scalar φ on an arbitrary control volume *V *with moving boundary is employed:(8)

where  is the flow velocity vector,  is the grid velocity of the moving mesh, Γ is the diffusion coefficient, *S*_φ _is the source term of φ and *∂V *is the boundary of the control volume *V*.

**Figure 4:**
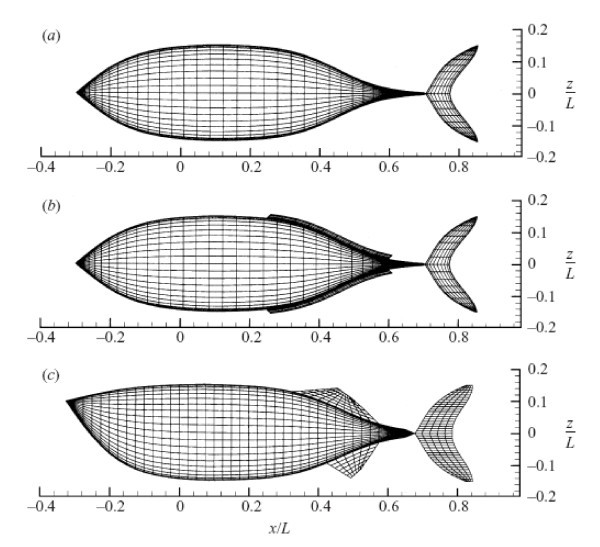
**Computational geometric forms of (a) the Robo Tuna, (b) tuna with dorsal/ventral finlets and (c) giant danio**. (From [26]).

**Figure 5:**
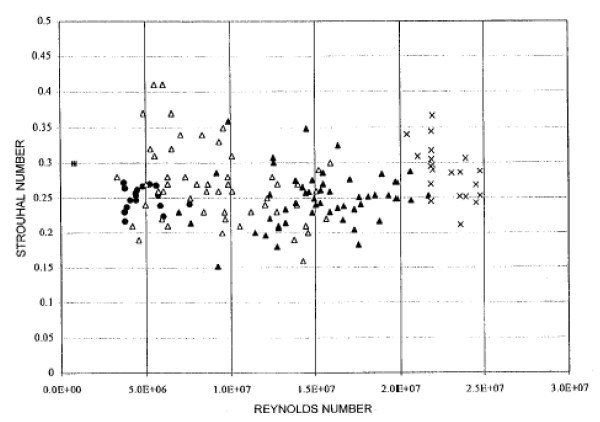
**Strouhal number for swimming dolphins as a function of Reynolds number**. (From Rohr et al. [27]).

The flow is characterized by spatially travelling waves of body bound vorticity. The mix between longitudinal and transverse flow features varies with the phase of oscillation and the unsteady velocity field varies throughout an oscillation cycle. The dynamic pressure distribution contour and the effect of the tail movement on the unsteady flow field of the fish-like body will show that there are high pressure zones at the rear of the body indicating strong vortex and turbulence. The kinematic parameters like Strouhal number, wavelength and oscillating frequency are based on the forward locomotion in a straight line with constant speed in the cruising direction. Figure 4 shows the computational geometric forms of (a) the Robo Tuna, (b) tuna with dorsal/ventral finlets and (c) giant danio [26]. Fish swimming kinematic data shows that the non-dimensional frequencies are close to the value predicted by the instability analysis. Figure 5, from Rohr et al. [27], shows Strouhal number as a function of the Reynolds number for numerous observations of trained dolphins with good agreement between theory and experiment.

Other example of using CFD to study biomimetic fluid flow problems include simulation of air flow around flapping insect wings, numerical simulation of electro-osmotic flow near earthworm surface and simulation of explosive discharge of the bombardier beetle.

**Figure 6:**
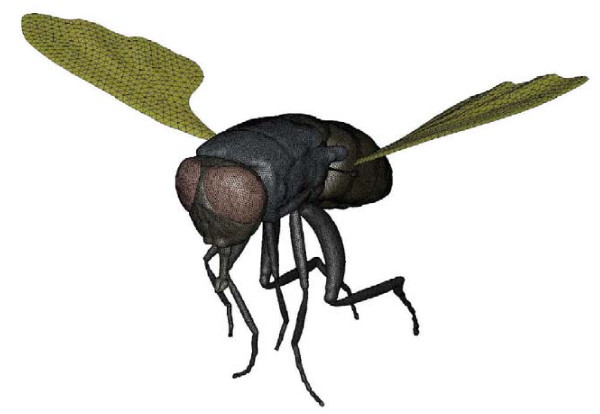
**Surface mesh on fly body**. (From [28]).

Kroger [28] made a CFD simulation study of air flow around flapping insect wings. The interest in the flapping-wing technique [29,30] is growing recently due to the fact, that the developments in micro-technology permit people to think about building very small and highly manoeuvrable micro-aircraft that could be used for search and rescue missions or to detect harmful substances or pollutants in areas that are not accessible by or too dangerous for humans. There are three basic principles that contribute to unsteady flapping-wing aerodynamics: delayed stall, rotational circulation and wake capture. However, the exact interactions between them are still subject to ongoing research by CFD simulation. Figure 6 shows surface mesh on fly body.

The dynamic mesh CFD model is used to examine critical flight simulations of normal aircraft, like the undercarriage lowering at low air speed, or the movement of sweep wings of fighter jets at high air speed. Next to flight applications, the dynamic mesh model can also simulate moving heart valves in the biomedical area, or small flapping membrane valves in micro-fluidics or the flow around any arbitrary moving part in other industry or sports applications.

**Figure 7:**
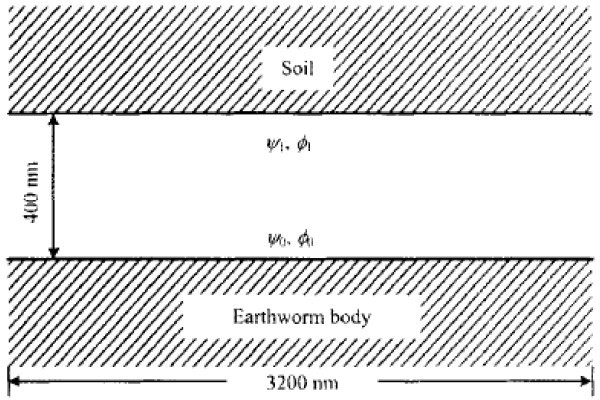
**Electroosmotic flow field between the surfaces of soil and earthworm**. (From [31]).

The electro-osmotic flow controlled by the Navier-Stokes equations near an earthworm surface has been simulated by Zu and Yan [31] numerically to understand the anti soil adhesion mechanism of earthworm. A lattice Poisson method (LPM), which is a derived form of LBM, has been employed to solve externally applied electric potential φ and charge distributions in the electric double layer along the earthworm surface. The external electric field is obtained by solving a Laplace equation. The simulation [32-35] showed that moving vortices, contributing to the anti soil adhesion, are formed near earthworm body surface by the non-uniform and variational electric force acting as lubricant. Figure 7 shows the electro-osmotic flow field between the surfaces of soil and earthworm.

**Figure 8:**
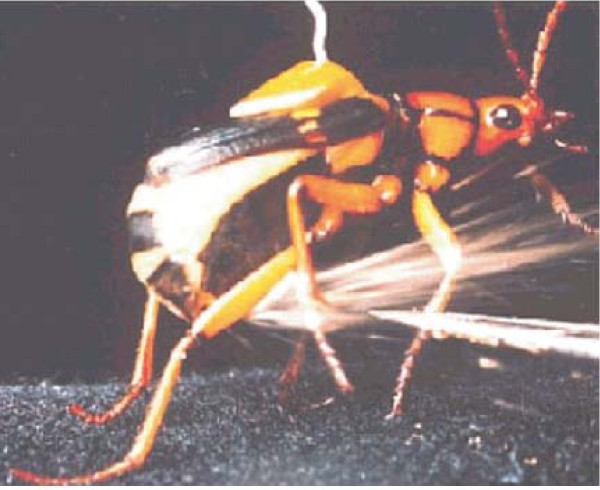
**A bombardier beetle ejecting its water-steam jet**. (From [36]).

A biomimetic CFD study [36-39] of the bombardier beetle's explosive discharge apparatus and unique natural 'combustion' technique in its jet-based defence mechanism helps designing a short mass ejection system and a long range of spray ejection pertinent to reigniting a gas turbine aircraft engine which has cut out, when the cold outside air temperature is extremely low. The beetle can eject a hot discharge to around 200 to 300 times the length of its combustor. Figure 8 shows a bombardier beetle (brachina) ejecting its water-steam jet at 100°C forward from the tip of its abdomen (from left to right).

### Hybrid molecular-continuum fluid dynamics simulation

Nanoscale systems such as GaAsMESFETs and SiMOSFETs semiconductor devices, ultra-fast (picoseconds or femtoseconds) pulsed lasers do not conform to the classical Fourier heat diffusion theory in which the mean free path of the energy carriers becomes comparable to or larger than the characteristic length scale of the particle device/system or the time scale of the processes becomes comparable to or smaller than the relaxation time of the energy carriers. Although numerical techniques like Boltzmann transport equation (BTE) or atomic-level simulation (MD) and Monte Carlo simulation (MCS) can capture the physics in this regime, they require large computational resources. The *C*-*V *hyperbolic equation, which is not subject to the Fourier law assumption of infinite thermal propagation speed, is also not free from anomalies.

#### Limitations of continuum description of a system

Finite difference and finite element methods serve well for continuum description of a system governed by a set of differential equations and boundary conditions. However, the problem arises when the system has atomic fabric of matter such as in the case of friction problems and phase-change problems of fluid freezing into a solid or dynamic transition such as intermittent stick-slip motion [40].

#### The molecular dynamics (MD) method

When a system is modelled on the atomic level such as in case of MD, the motion of individual atoms or molecules is approximated. The particle motion is controlled by interaction potentials and equations of motion. MD is used for systems on the nanometre scale.

#### Coupling MD-continuum

Coupling two very different descriptions of fluids at MD-continuum interface is a serious issue. The overlapping region of two descriptions must be coupled over space as well as time giving consistent physical quantities like density, momentum and energy and their fluxes must be continuous. Quantities of particles may be averaged locally and temporally to obtain boundary conditions of continuum equations. Getting microscopic quantities from macroscopic non-unique ensembles is, however, difficult.

#### Coupling schemes

Several coupling schemes [40-44] have been developed and the two solutions relax in a finite overlap region before they are coupled. Equations of motion are the language of particles and these are coupled with the continuum language, i.e. the differential equations. The coupling mechanism transmits mass flux, momentum flux and energy flux across the domain boundary. If the remaining boundaries are sealed, i.e. the simulated system is closed; the coupling ensures conservation of mass, momentum and energy.

The two domains are coupled to each other by ensuring that the flux components normal to the domain boundary match. If particles flow towards the boundary, a corresponding amount of mass, momentum and energy must be fed into the continuum. Conversely, any transport in the vicinity of the boundary on the part of the continuum must provide a boundary condition for transport on the part of the particles.

**Figure 9:**
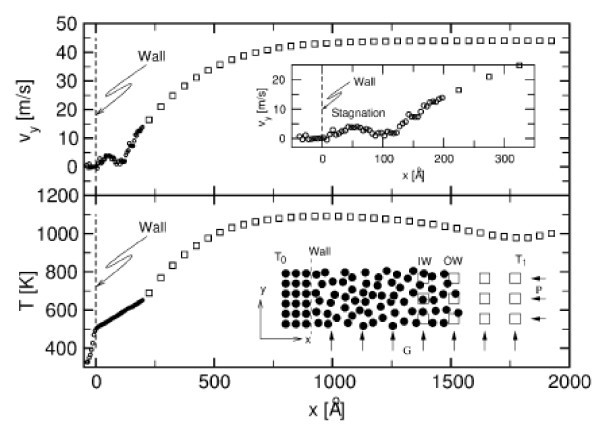
**Plot of velocity parallel to a macroscopically flat wall and of temperature as a function of wall distance**. Spheres and squares represent the particle and the continuum domain, respectively. (From [40]).

Figure 9 shows the velocity and temperature profiles observed in a simulation using Lennard-Jones particles and a Navier-Stokes continuum.

#### Smoothed particle hydrodynamics

Sousa [45] presented a scientific smoothed particle hydrodynamic (SPH) multiphysics simulation tool applicable from macro to nanoscale heat transfer. SPH [45] is a meshless particle based Lagrangian fluid dynamic simulation technique; the fluid flow is represented by a collection of discrete elements or pseudo particles. These particles are initially distributed with a specified density distribution and evolve in time according to the fluid heat, mass, species and momentum conservation equations. Flow properties are determined by an interpolation or smoothing of the nearby particle distribution with the help of a weighting function called the smoothing kernel. SPH is advantageous in (1) tracking problems dealing with multiphysics, (2) handling complex free surface and material interface, (3) parallel computing with relatively simple computer codes, (4) dealing with transient fluid and heat transport.

Following the original approach of Olfe [46] and Modest [47] in case of radiative heat transfer, Sousa [45] made the SPH numerical modelling for the ballistic-diffusive heat conduction equation. In this method, the heat carriers inside the medium are split into two components: ballistic and diffusive. The ballistic component is determined from the prescribed boundary condition and/or nanoscale heat sources and it experiences only outscattering; the transport of the scattered and excited heat carriers inside the medium is treated as diffusive component.

#### Intrinsic complex issues in hybrid method

**Figure 10:**
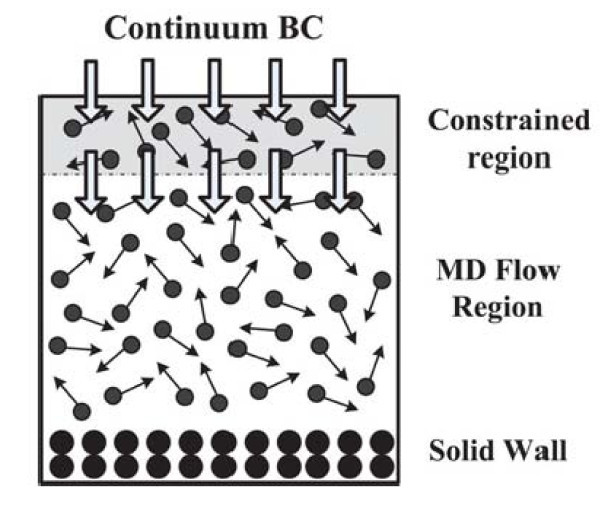
**Schematic representation of a molecular region in a hybrid simulation**. (From [48]).

The development and optimization of the performance of micro and nano fluidic devices requires numerical modelling of fluid flow inside micro and nanochannels. The nature of the phenomena involved in these devices invariably and predominantly has the interfacial interactions because of high surface-to-volume ratio and is characterized by an inherent multiscale nature [48-62]. The traditional continuum models do not capture the flow physics inside the micro and nano scale systems because they neglect the microscopic mechanisms at these scales. The MD is a microscopic model and this can be used where macroscopic constitutive equations and boundary conditions are inadequate. Figure 10[48] shows the schematic representation of a molecular region in a hybrid simulation. The MD are well suited for the study of slip generation in the solid-fluid interface and other surface properties like nanoroughness and wettability and the boundary conditions. However, high computational cost restricts the molecular simulations to their applications to nanoscale systems and time scales below microseconds. This disparity of spatial and temporal scales is overcome in the hybrid atomistic-continuum multiscale frameworks where the molecular description models only a small part of the computational domain, since the physics of this part of the system cannot be represented by the continuum model. The boundary condition is transferred accurately and efficiently between the atomistic and continuum description in the hybrid methods. Since the microscopic description requires more degrees of freedom than the macroscopic one, the transfer of macroscopic information on a molecular simulation becomes all the more a challenging task.

#### MD model and the Maxwell-Boltzmann velocity distribution

The MD atomistic model in the micro-scale framework is a deterministic method. In this model, the evolution of the molecular system is obtained by computing the trajectories of the particles based on the classical molecular model. The continuum conditions can be applied to molecular domain either by the method based on continuous rescaling of atomic velocities or by the periodic resampling method of atomistic velocities that employs velocity distribution functions such as Maxwell-Boltzmann or Chapman-Enskog distribution for non-equilibrium situations of hybrid simulations in dilute gases employing geometrical decomposition and state coupling. The Maxwell-Boltzmann velocity distribution is the natural velocity distribution of an atomic or molecular system in an equilibrium state defining the probability of one-dimensional velocity components of an atom assuming a specific value based on temperature and the atomic mass. The reflective plane placed at the upper boundary of the boundary condition transfer region maintains every particle inside the molecular domain. This scheme is simpler than the velocity reversing scheme, but this can be applied only to incompressible flows because the normal pressure is a result of the reflected atoms.

#### Rescaling techniques

In the rescaling techniques, in addition to the velocity restrictions, the continuum pressure applies to the atomistic region. The normal pressure is applied through external forces generating a potential energy field. Energy is decreased because of the reduction of potential energy of the atoms moving towards the continuum boundary. The resulting energy oscillations in the molecular system are reduced by velocity reversing of the outermost atoms. This scheme is simple and robust because of uncontrolled transfer of energy. The continuum temperature to the molecular system is accomplished by an energy transfer scheme. The energy is added or removed from the microscopic system to parallel the macroscopic temperature without modifying the mean velocity of the particles. The energy transfer takes place independent of each dimension and is accomplished by the velocity vectors of the atoms [42,61-68].

#### Issues related to boundary conditions in hybrid multiscaling modelling

Drikakis and Asproulis [69] applied macroscopic boundary conditions in hybrid multiscale modelling. MD microscopic simulation was employed. They employed the methods for various liquid and gas flows with heat transfer and identified specific parameters for accuracy and efficiency. Their work has shown that knowledge about boundary conditions development and application is needed in multiscale computational frameworks. Continuum temperature and velocity as well as macroscopic pressure constrain molecular domain. Inconsistent pressure can shrink the simulation domain and the particles may drift away generating errors and instabilities in the hybrid procedure. Also, the size of the regions for the application of velocity constrains is important to avoid unrealistic heat transfer across the computational domain and inconsistencies between the molecular and continuum state. Resampling frequency and the termination of the atomistic region have significant impact in the resampling techniques and these can influence trapping of particles in the constrained region and may cause deviations between the macroscopic and microscopic velocities. The domain termination needs correct continuum pressure application.

### Challenge in biomimetic flow simulation

The task of imitating biological functional surfaces with variety of complex three-dimensional micro- and nano-structures is very challenging in biomimetic flow simulation. The transfer of biological morphologies of plants and animals by imitating both geometrical and physical similarity to technological applications is to be identified [70-127]. Studies on micro surface structures of different species are to be made by scanning electron microscope (SEM) and atomic force microscope (AFM) to imitate engineering functional surfaces. The mesoscopic LBM has been applied in studying electro-osmotic driving flow within the micro thin liquid layer near an earthworm body surface [128]. The moving vortices give the effect of anti soil adhesion. Few multiphase LBM models are the pseudo-potential model, the free energy model and the index-function model [129-132]. In LBM, effective interaction potential describes the fluid-fluid interaction. Interface is introduced by modelling the Boltzmann collision operator imposing phase separation. Also, the fluid-fluid interactions are represented by a body force term in Boltzmann equation. In this case, second-order terms in the pressure tensor are removed and more realistic interfacial interactions are produced.

Hard spheres fluids, square well fluids and Lennard-Jones fluids are model fluids in MD. The fluid flow and heat transfer in micro-scale and nano-scale systems get microscopic and nanoscopic insight from MD [133].

## Conclusions

A comprehensive and state-of-the-art review of CFD techniques for numerical modelling of some biomimetic flows at different scales has been done. Fluid-fluid interfaces contacting with functional solid surfaces have been discussed. The multiphysics modelling at different scales by Navier-Stokes and energy equations, mesoscopic LBM, MD method and combined continuum-MD method with appropriate coupling schemes have been dealt with in detail.

## Abbreviations

AFM: atomic force microscope; BTE: Boltzmann transport equation; CFD: computational fluid dynamics; DSMD: direct simulation of Monte Carlo; FEM: finite element method; FVM: finite volume method; HPC: high performance computer; LBM: lattice Boltzmann method; LPM: lattice Poisson method; MCS: Monte Carlo simulation; MD: molecular dynamics; RANS: Reynolds-averaged Navier-Stokes; SEM: scanning electron microscope; SPH: smoothed particle hydrodynamic; TRIZ: Teoriya Resheniya Izobretatelskikh Zadatch.

## Competing interests

The authors declare that they have no competing interests.

## Authors' contributions

All authors read and approved the final manuscript.
